# The Isoform Selective Roles of PI3Ks in Dendritic Cell Biology and Function

**DOI:** 10.3389/fimmu.2018.02574

**Published:** 2018-11-15

**Authors:** Ezra Aksoy, Loredana Saveanu, Bénédicte Manoury

**Affiliations:** ^1^Centre for Biochemical Pharmacology, William Harvey Research Institute, Bart's and the London School of Medicine Queen Mary University of London, London, United Kingdom; ^2^Institut National de la Santé et de la Recherche Médicale, Unité UMR 1149, Centre de Recherche sur l'Inflammation, Paris, France; ^3^Université Paris Diderot, Faculté de Médecine Xavier Bichat, Paris, France; ^4^Institut Necker Enfants Malades, Institut National de la Santé et de la Recherche Médicale, Unité 1151, Paris, France; ^5^Centre National de la Recherche Scientifique, Unité 8253, Paris, France; ^6^Université Paris Descartes, Sorbonne Paris Cité, Faculté de Médecine Paris Descartes, Paris, France

**Keywords:** dendritic cell, antigen presentation, PI3K, toll like receptors, phospholipids

## Abstract

Phosphoinositide-3 kinases (PI3Ks) generate 3-phosphorylated phosphoinositide lipids that are implicated in many biological processes in homeostatic states and pathologies such as cancer, inflammation and autoimmunity. Eight isoforms of PI3K exist in mammals and among them the class I PI3K, p110γ, and PI3Kδ, and class III Vps34 being the most expressed and well characterized in immune cells. Following engagement of pathogen recognition receptors (PRRs), PI3Ks coordinate vital cellular processes of signaling and vesicular trafficking in innate phagocytes such as macrophages and professional antigen presenting dendritic cells (DCs). Although previous studies demonstrated the involvement of PI3K isoforms in innate and adaptive immune cell types, the role of PI3Ks with respect to DC biology has been enigmatic. Thus, this review, based on studies involving PI3K isoforms, highlight how the different PI3Ks isoforms could regulate DC functions such as antigen processing and presentation including PRR responses.

## Introduction

**Figure 1:**
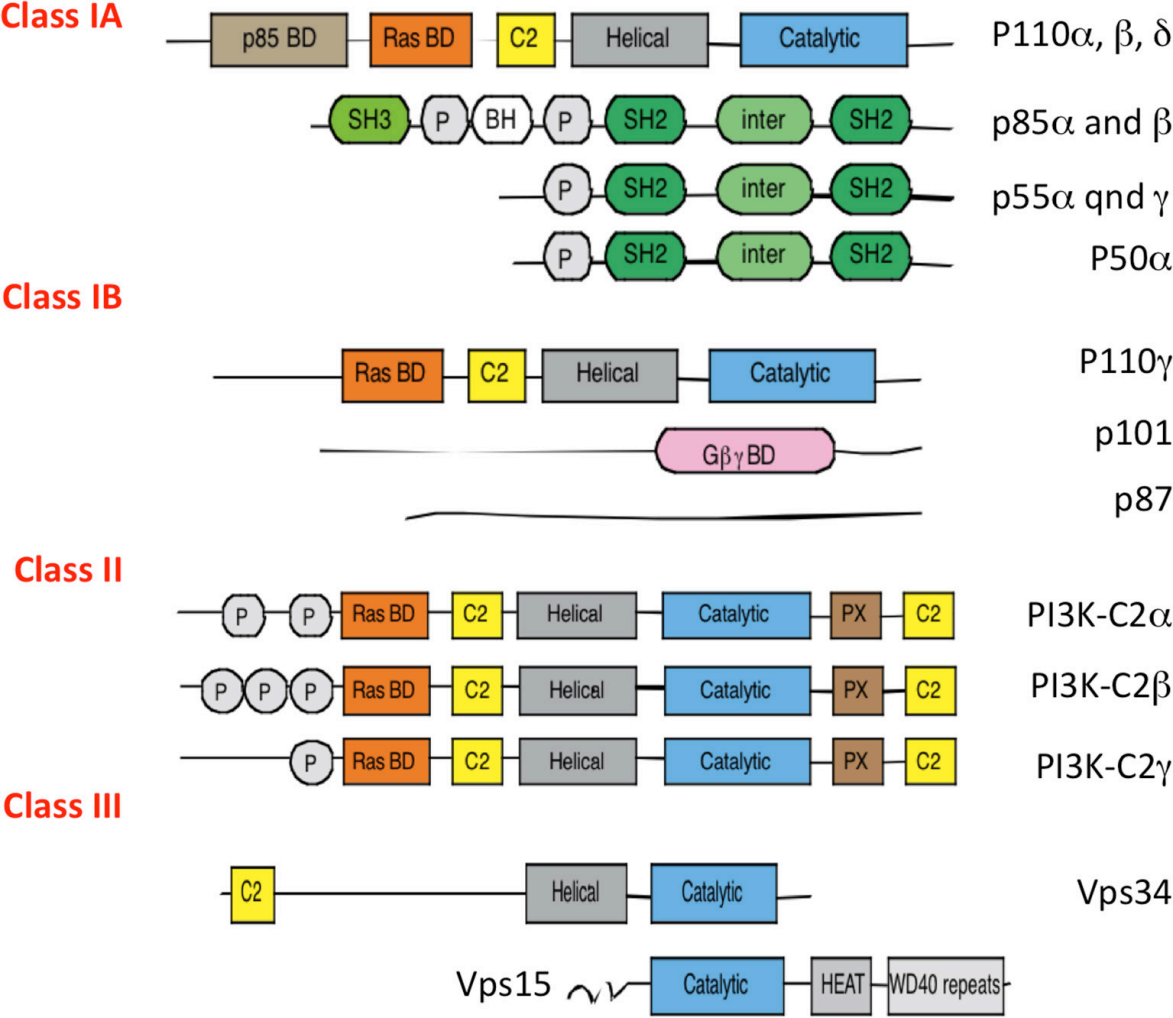
Different classes of PI3Ks. All PI3Ks isoforms (p110 α, β, δ, γ, C2α, C2β, C2γ, Vps34) have 3 to 5 domains: an N terminal domain which can bind the regulatory p85 proteins, a Ras binding domain, a C2 domain which binds membranes, an helical domain of unknown function and a catalytic subunit with kinase activity. They associate with a regulatory subunit, p85 isoforms (for p110 α, β, δ), p101, and p87 for p110γ and Vps15 for Vps34. P85 regulatory subunits are encoded by different genes: PI3KR1 (depending on the promoter will give p85α, p55α, p50β), PI3KR2 (p85β), and PI3KR3 (p55γ). PI3Ks use phospatidylinositol lipids as their substrates.

PI3Ks are activated by diverse signaling pathways including small G proteins of Ras and Rac family, tyrosine kinases- or G-protein- coupled receptors (1). PI3Ks comprise three class I, II, and III family of enzymes. Except for class II PI3K (isoforms α, β, δ) which has only catalytic subunits, class I (isoforms α, β, δ, γ) and class III (Vps34) PI3Ks form heterodimeric molecules, which consist of the assembly of a catalytic and a regulatory subunit(s) (Figure 1). PI3Ks phosphorylate the 3-hydroxyl group of the inositol ring of three species of phosphatidylinositol (PI) lipid substrates; namely, PI, PI-4-phosphate PI(4)P, and PI-4,5-bisphosphate PI(4,5)P_2_. They catalyze the formation of 3-phosphorylated phosphoinositide lipids, such as phosphoinositol-4,5 biphosphate PI(4,5)P_2_ into phosphoinositol-3,4,5 triphosphate PI(3,4,5)P_3_ for class I, and generation of phosphoinositol triphosphate PI(3)P from phosphoinositol (PI) or phosphoinositol-3,4 bisphosphate PI(3,4)P_2_ from phosphoinositol phosphate PI(4)P for class II, and finally only the production of phosphoinositol triphosphate PI(3)P from PI for class III.

**Figure 2:**
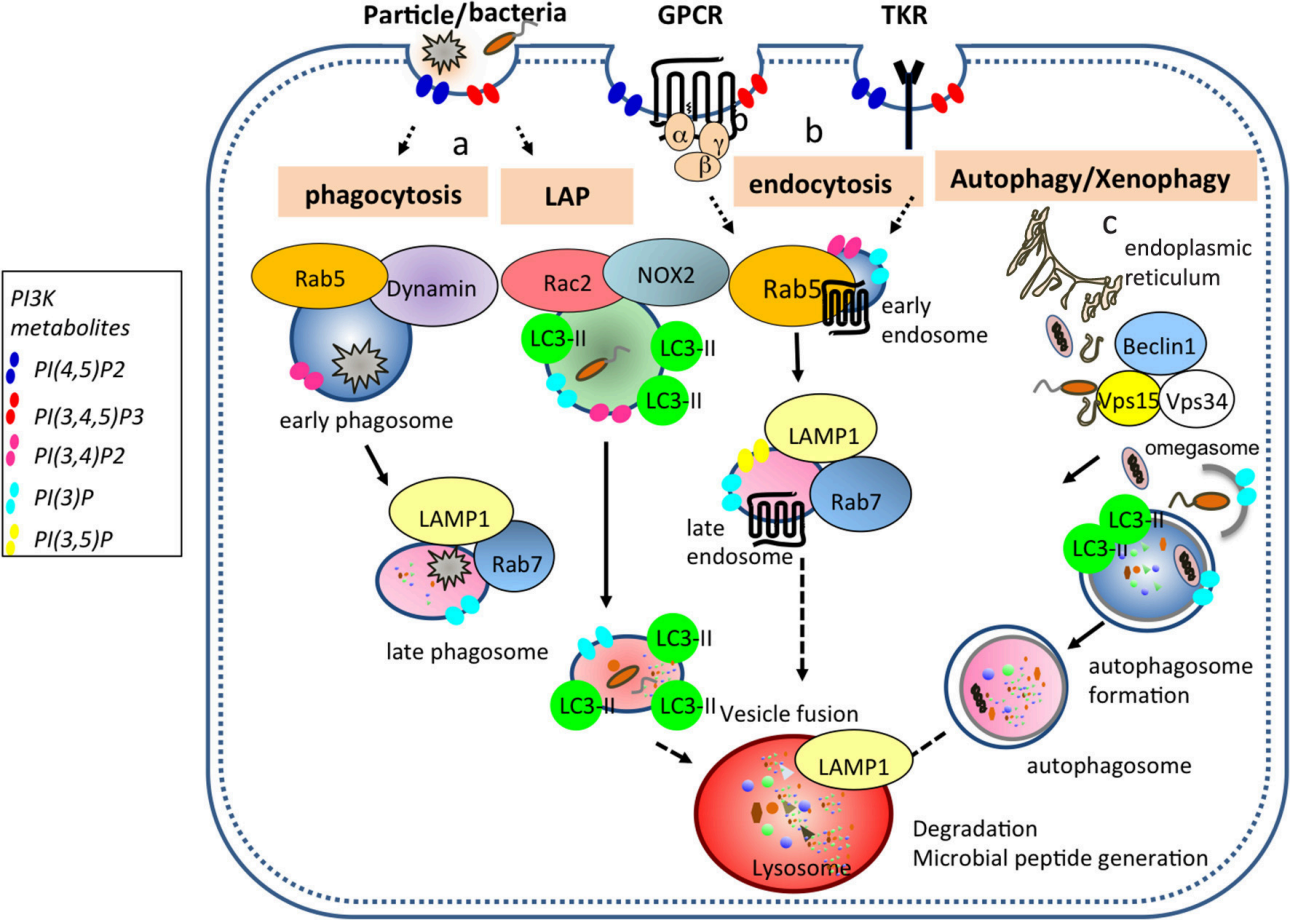
PI3Ks generate membrane phospholipids important for vesicular trafficking events in DCs. (a) Bacteria, cell debris, and large particles are taken up in Rab5^+^ dynamin^+^ early phagosomes that undergo maturation to generate Rab7^+^ LAMP1^+^ late phagosome. Pathogens such as bacteria and yeast can be internalized into LC3 associated phagosome (LAP) where Rac2 and NOX2 are recruited and are required to generate reactive oxygen species (ROS). (b) During endocytosis, ligands bound to G protein coupled- and tyrosine kinase-receptors are taken up from the plasma membrane into Rab5^+^ early endosomes and then either traffic to Rab7^+^ LAMP1^+^ late endosomes or are recycled back to the plasma membrane. (c) During autophagy, a double membrane organelle is generated from an omegasome (specific ER structure) where Vps34, Vps15, and Beclin1 are recruited. This double membrane organelle will form later the autophagosome by ingesting cytoplasmic material. Ultimately, all transport vesicles (late phagosome, late endosome, autophagosome) will fuse with the lysosomes for cargo degradation. PI(4,5)P_2_ is enriched proximal to the plasma membrane and in lysosomal location. Class I PI3Ks produce PI(3,4,5)P_3_ from PI(4,5)P_2_, while class II and III PI3Ks produce PI(3)P following phagosome enclosure, and PI(3,4,5)P_3_ is converted into PI(3,4,)P_2_ by SHIP1 and SHIP2 (3) phosphatases. PI(3)P and PI(3.5)P_2_ are present in late endosomes.

PI3Ks are evolutionarily conserved from soil dwelling amoeba, *Dictyostelium discoideum* to mammals (2). The evolutionary conservation of PI3K families and its functions from the *Dictyostelium*, an archetypical phagocyte, to mammals in generating membrane phospholipids highlights the importance of these kinases in endocytic and phagocytic processes and their non-redundant functions in innate phagocytes including dendritic cells (DCs, Figure 2).

## The role of PI3Ks in DC mediated homeostatic regulation of inflammation and immunity

PI3K isoforms, particularly class I family of enzymes, have distinct tissue and cell distribution in mammals. Whereas PI3Kδ and PI3Kγ are preferentially expressed in immune cells of hematopoietic origin at high levels, PI3Kα, and PI3Kβ are ubiquitous and broadly expressed in all somatic cells (4). However, data from published microarrays suggest differential expression of PI3Kγ and δ in subset of DCs. PI3Kδ mRNAs are well expressed in plasmacytoid DCs (pDCs), subset of DCs producing high amount of type I interferon (IFN) following viral infection, and PI3Kγ mRNA expression was found exclusively restricted to type 2 DCs or cDC2 (5). These data remain to be confirmed at the protein level but might imply a preferential role for PI3Kγ in major histocompatibility class II (MHCH II) antigen presentation as cDC2 are the main DCs subset to present antigens to CD4^+^ T cells (6).

Over the last decade, a number of PI3K isoform-selective gene-targeted mouse models for class I PI3K catalytic and regulatory subunits and class III PI3K have been generated and together with the development of isoform specific inhibitors have greatly advanced the understanding of PI3K signaling in mammalian biology. Due to the cell type specific expression of PI3Kγ and PI3Kδ, targeting these isoforms affect innate and adaptive immune responses (7). Studies using genetic and pharmacological targeting of PI3Kδ isoform has shown PI3Kδ is a homeostatic regulator of activation, downstream of Mal-MyD88-coupled TLR2 and TLR4 signaling pathways in DCs. PI3Kδ achieves this by dampening pro-inflammatory cytokine secretion, while supporting production of IL-10 (8). PI3Kγ was shown to be needed for the development of lung CD11b^+^DC and CD103^+^DCs especially by regulation of signaling downstream of Flt3, whereas it was dispensable for DC development in many other tissues (9). PI3Kγ deficiency was, in mice, demonstrated to increase susceptibility to influenza virus infection due to impaired T cell priming by lung resident DC and delayed viral clearance owing to the pre-existing DC developmental deficiency in the lung compartment. In another study, PI3Kγ deficient mice showed selective reduction in the number of skin Langerhans cells and in lymph node CD8α^−^DC (10). Pan PI3K inhibitors such as wortmannin and LY294002 and deficiency in the PI3Kδ or p85α regulatory isoform of class I PI3Ks (that couples to PI3Kα, β or δ isoforms) were found to enhance TLR4 mediated pro-inflammatory responses by LPS in myeloid cells including DC and macrophages (8, 11–15). TLR4 activation triggers Mal-MyD88 signaling originating from the plasma membrane via phosphoinositol 4,5-bisphosphate [PI(4,5)P_2_]-localized TIRAP signaling, which is consecutively followed by phosphoinositol–binding TRAM mediated endocytic pathway leading to type I IFNβ production (16, 17). This study has shown that PI3Kδ mediates the switch between TIRAP-dependent pro-inflammatory pathway coupled to TLR4 endocytosis and TIRAP degradation subsequently leading to TRAM-dependent type I IFNβ and IL-10 secretion (8). This type of homeostatic control indicates that PI3Kδ signaling pathway is a physiological regulator of inflammation by dampening endotoxemia and sepsis.

While PI3Kγ and PI3Kδ roles in TLR mediated pro-inflammatory reactions has been extensively addressed, their role in DC antigen presentation and DC-dependent T cell-mediated immunity in infection models have yet to be examined. Indeed, a recent study, reporting the diminished ability of WT OT1 T cells to provide help for the p110δ kinase-deficient T cells in *Listeria* expressing ovalbumin (OVA*)* infection model, indicates a possible role of DCs supporting antigen-specific CD8^+^ T cell expansion in a PI3Kδ dependent manner (18). In this study, WT OT1 cells, injected into p110δ^D910A^ hosts, showed reduced primary immune responses and proliferation. Likewise in other studies, the PI3Kδ and p85α regulatory subunit deficient mouse strains were found to exhibit enhanced resistance to *Leishmania major* infection, despite mounting impaired T cell responses and yet intact or enhanced DC pro-inflammatory cytokine response (19). Also, DCs lacking SHIP1, the phosphatase converting PI(3,4,5)P_3_ into PI(3,4)P_2_, were able to mature and induce autoimmunity by promoting CD8^+^ T cells expansion and INFγ production in an *in vivo* model of diabetes (20). In agreement with this, SHIP1 overexpression led to an inability of DCs to trigger T cell auto immunity (20) suggesting that PI regulated by SHIP phosphatases and PI3Ks play a major role in DCs antigen presentation.

Recently, mice deficient for Class III PI3K or Vps34 in CD11c^+^DCs were generated (21). These mice showed a specific reduction in the number of CD8^+^DCs, subset of DCs specialized in MHC class I (MHCI) antigen cross presentation, in the spleen and were defective at presenting dying cell-associated antigens to the MHCI antigen cross presentation pathway. The defect was linked to a reduced expression of Tim4, a phospatidylserine receptor require for uptake of apoptotic cells, in CD8^+^DCs lacking Vps34. In contrast, presentation of antigens by the classical MHC class I and II pathways was increased and might be linked to an overall enhancement of DCs activation at the steady state in the Vps34-CD11c^+^DCs deficient mice (21). In addition, a highly selective and potent class III PI3K inhibitor, SAR405 was reported to influence vesicle trafficking and autophagy (22) and it will be of importance to unravel the exact role of Vps34 kinase and scaffolding functions regulating DC biology.

The PI3Kδ and PI3Kγ isoforms are key targets, being harnessed in chronic inflammatory and autoimmune conditions such as asthma, psoriasis rheumatoid arthritis (RA) and systemic lupus erythematous (SLE), with single or dual inhibitors for both isoforms being tested in clinical trials (23). In OVA-induced allergic inflammation models, genetic or pharmacological targeting of PI3Kδ was reported to reduce inflammatory cell infiltrates and IL-17 secretion (24), while PI3Kδ deficiency in mice resulted in suppression of Th2 cell mediated responses to OVA following immunization with OVA antigen *in vivo* (25). Although mice lacking PI3Kγ exhibited reduced levels of eosinophilic airway inflammation, they did not show significant differences in serum OVA-specific IgE and IgG1 levels and CD4/CD8 T cell balance (26). However, PI3Kδ deficient animals display reductions in the levels of eosinophil recruitment and Th2 cytokine response, indicating that DC-mediated antigen priming of T cells might be altered under PI3Kδ deficiency, a topic unaddressed so far (26).

## The role of PI3Ks in DC migration

The activation of class I PI3K downstream of several receptors for chemo attractants, such as chemokines, complement component C5a, Nformylmethionyl- leucyl-phenylalanine and sphingosine-1-phosphate explains the pivotal role of these enzymes in cell migration (27). Interestingly, the role of class I PI3K-dependent signaling in migratory responses to chemokines was mainly explored in leukocytes, neutrophils and to a lesser extent, in macrophages (28–31). Nevertheless, it is very likely that class I PI3K signaling, especially PI3Kγ and PI3Kδ, have also crucial roles in DCs migration, a process necessary for DCs to reach secondary lymphoid organs to present antigens to T cells, in a similar way as they act in neutrophils and macrophages. The major class I PI3K activated downstream chemokine receptors in neutrophils is considered to be the class IB PI3Kγ, although an important degree of cooperation, still incompletely understood, exists between PI3Kγ and PI3Kδ in the control of neutrophil migration. Thus, early steps of neutrophil migration depend on PI3Kγ, while late steps of neutrophil long-term migration requires the cooperative action of PI3Kγ and PI3δ (32).

The central molecular event connecting PI3K activation with cell migration is the production of PI(3,4,5)P_3_, which interacts with proteins containing pleckstrin homology domains (PH). Proteins containing PH-domains include several master regulators of cytoskeleton remodeling that are essential for cell migration. These are the guanine nucleotide exchange factors for Rac (P-REX1 and 2 and VAVs 1, 2 and3) (33, 34) that regulates F-actin polymerization and myosin assembly (35). The biosensors, formed by GFP fusion with typical PH domains, specific for PI(3,4,5)P_3_, which allows the visualization of PI(3,4,5)P_3_ in cells clearly established that PI(3,4,5)P_3_ is concentrated to the leading edge of the cell and this is mandatory for Rac activation, cytoskeleton rearrangements and finally, orientated neutrophil migration (36, 37). Indeed, neutrophil migration and wound repair were enhanced in a zebrafish model where SHIP phosphatases were depleted (38) but were restored to control level when cells deficient for SHIP were incubated with PI3Kγ inhibitor. Whether a similar role of PI3Kγ-mediated distribution of PI(3,4,5)P_3_ occurs during DC migration remains unknown. However, studies involving experimental dextran sodium sulfate (DSS)-induced colitis reported that genetic or pharmacological inhibition of PI3Kγ is protective, which may be in part due to inhibitory effects on inflammatory leukocyte migration.

Investigation of the role of PI3Kγ and PI3Kδ in DC and DC-like cell lines migration is an important issue, which should be studied in the future. Significant layers of complexity exist in the study of class I PI3K, notably PI3Kγ and PI3Kβ, in DC migration. One is the diversity of DC populations and their different roles in the immune response (39), which arise the possibility that the inhibition of one member of class I PI3K will affect the migratory and chemotactic abilities of a particular DC subset. Thus, it would be interesting to investigate if pDC migration is depending on PI3Kδ, while cDC2 migration depends on PI3Kγ (5). PI3Kγ was shown to play a key role in DC trafficking and in the activation of specific immunity since PI3Kγ deficient DCs show inhibited migration to the lymph nodes (LN) in response to CCR7, which was correlated with reduced DC numbers in LNs (10). Another level of complexity is given by the existence of two regulatory sub-units for the master PI3K kinase involved in immune cell migration, the PI3Kγ (class IB). Unlike all other class I PI3K, this enzyme has two regulatory subunits, both of them expressed, but never studied in DCs. It remains to be investigated if these two regulatory subunits of PI3Kγ have similar functions in DC migration, by means of differential coupling to distinct signaling receptors. And, as a final level of complexity of the class I PI3K involvement in DCs migration, it should be underlined that essential regulators of Rac activity, such as Vav1, 2, 3 and P-Rex1, 2 are differentially expressed in DC subsets according with the mRNA expression data (5). Thus, Vav1 and 2 proteins are expressed mainly in pDCs, Vav3 in type 1 DCs, while type 2 DCs express P-Rex 1 and 2 (5). This pattern of Rac GEFs expression suggest that downstream targets of class I PI3K activation might also contribute to different molecular pathways regulating DC migration in a subset specific way.

## PI3K role in DC tolerogenic functions and their antigen presentation potential

Although the exact roles of PI3K isoforms in antigen processing and presentation in DCs remains unknown, there are indications that tolerogenic DC functions may rely on PI3K-Akt signaling. PI3K/Akt/mTOR pathway is central to the regulation of glycolytic metabolism (40, 41), and equally important in DC immunometabolic activities and maturation associated increase of co-stimulatory molecules and MHCII surface expression (42). Consistent with this, PI3K/Akt axis was shown to be essential for sustained commitment to glycolysis in TLR activated DCs (43). Furthermore, the existence of close links between glycolysis-hypoxia and PI3K-Akt signaling in immune cells indicated that hypoxic conditions may influence PI3K signaling and thus impact DC functions involving their migration capacity and their ability to induce Tregs in DSS model of colitis as reported (44, 45). This has come to attention since a recent work using human 1,25D3-DCs demonstrated that tolerogenic DCs, generated by 1,25(OH)2D3, rely on glucose accessibility and aerobic glycolysis to maintain their tolerance-inducing properties (46). Indeed, inhibition of the PI3K/Akt/mTOR pathway was shown to reverse the tolerogenic function of 1,25(OH)2D3-modulated DCs by a transcriptional reprogramming of glycolysis associated genes. Furthermore, pan PI3K inhibitors and rapamycin were found to hinder tolerogenic DCs function, in part, by partial restoration of CD4^+^ T cell proliferation (46).

PI3Kδ and PI3Kγ single and dual isoform selective inhibitors and mice deficient in p110δ or p110γ do not manifest overt pathogenic phenotypes, despite exhibiting a wide spectrum of immunological defects. Nevertheless, patients treated with the PI3Kδ-selective inhibitor idelalisib (Zydelig) manifest serious side effects, which results in colitis, diarrhea, neutropenia, pneumonitis and some level of liver damage (47, 48). This overt clinical phenotype in humans remarkably coincides with the occurrence of spontaneous colitis in PI3Kδ deficient mice (49). These mice progressively develop colitis (49) which depends on the presence of enteric microbiota for colitis development in germ free p110δ deficient animals (50, 51). The cellular and molecular mechanisms for increased colitis susceptibility, under PI3Kδ deficiency requires careful examination of the host immune status both in mouse studies and in human trials (52). PI3Kδ deficiency may increase susceptibility to common infections due to defects in mounting T cell immunity, not only owing to the diminished function of PI3Kδ deficient T cells, but also because of faulty function of DC in peripheral tissues, enriched in microbiome. A clear dissection between the T cell- and DC-intrinsic role of PI3Ks in mounting the adaptive immune response require in the future the generation of tissue-specific PI3K-deficient mouse strains.

## PI3K role in DC-mediated cancer immunity

PI3K pathway-targeted therapies have been tested in oncology trials and several pharmaceutical companies have developed selective PI3K inhibitors to target PI3K pathway in diverse types of cancer cells. Due to the restricted tissue and cell expression, PI3Kδ and PI3Kγ are attractive drug targets in hematological cancers, and a distinguished success of efficacy was reported with PI3Kδ-selective idelalisib in treating Chronic Lymphocytic Leukemia (CLL) and non-Hodgkin's lymphoma, and idelalisib is now approved for clinic (47, 48, 53).

Evidence from genetic or pharmacological targeting studies indicate that inhibiting class I PI3K isoforms may be beneficial in improving cancer immunotherapy (54, 55). In parallel to PI3K research in oncology, several studies have uncovered exciting and unexpected roles for PI3K catalytic and regulatory subunits in cancer immunity, potentially by boosting the efficacy of PI3K-targeted therapies by adapting the immune compartment (1). Previously, genetic or pharmacological inhibition of PI3Kδ reduced tumor burden and metastasis in a range of mouse cancer models including melanoma, thymoma, lung, and breast cancer (54). In these models, PI3Kδ inhibition was found to attenuate Treg function and tumor infiltration and surprisingly did not alter cytotoxic T cell responses, resulting in enhanced anti-tumor immunity. Interestingly, one of the common side effect of checkpoint inhibitors in cancer therapy using PDL1, PD-1, and CTLA blocking antibodies concerns colitis development due to inhibition of Treg functions (56, 57). It is plausible that combined with Treg functions, DC-mediated tolerance induction may be altered in PI3Kδ deficiency in both humans and mice.

Genetic or pharmacological inhibition of p110γ was reported to reduce tumor growth and metastasis in melanoma, lung, pancreatic breast, and colon cancer models. The efficacy of inhibiting PI3Kγ signaling was suggested to involve myeloid cell recruitment to the tumor microenvironment through integrin α4β1 mediated adhesion in response to chemotactic signals. Therefore, intervention of p110γ signaling appears as an effective target in reducing tumor-associated inflammation and subsequent angiogenesis response (58, 59).

Overall based on a number of studies, isoform selective PI3K inhibition in cancer therapy appears to be efficacious, but it will be critical in the near future to uncover DC-intrinsic roles of PI3K isoforms, particularly in the context of antigen presenting cells (APC) functions, since DC orchestrate immune responses by activating antitumor immunity. Because PI3K inhibitors, particularly PI3Kδ inhibitors, have been reported to boost proinflammatory TLR responses and IL12p70 production, it will be interesting to find out whether APC functions and Th1 inducing capacity of DCs will be affected under PI3K inhibition. Understanding the role of PI3K inhibition in both innate and adaptive immune functions of DCs will indicate if PI3K isoform selective inhibitors may be utilized as novel “innate” check point inhibitors to boost current cancer therapies.

## Conclusion

Overall, the exact role and contribution of PI3K isoforms in APC function of DC in T cell priming is still enigmatic and new tools such as tissue-specific PI3K-deficient mouse strains should be developed in the future. These will allow underpinning DC-intrinsic roles of PI3K isoforms in antigen presentation during cancer, auto inflammatory and autoimmune diseases.

## Author contributions

EA, LS, and BM wrote the manuscript. EA and BM contributed critically in the conceptualization and arrangement of the review.

### Conflict of interest statement

The authors declare that the research was conducted in the absence of any commercial or financial relationships that could be construed as a potential conflict of interest.

## Abbreviations

APC: antigen presenting cells
PI3Ks: Phosphoinositide−3 kinases
PRRs: pathogen recognition receptors
DC: dendritic cells
PI: phosphatidylinositol
PI(4)P: phosphoinositol phosphate
PI(4,5)P_2_, phosphoinositol-4: 5-bisphosphate
PI(3,4,5)P_3_, phosphoinositol-3,4: 5 triphosphate
PI(3,4)P_2_, phosphoinositol-3: 4 bisphosphate
PI(3)P: phosphoinositol triphosphate
IFN: interferon
MHCI: major histocompatibilty class I
pDCs: plasmacytoid DCs
OVA: ovalbumin
MHCII: major histocompatibility class II.
